# Probing biomolecular condensates with a minimally perturbative experimental readout framework

**DOI:** 10.1042/BCJ20260173

**Published:** 2026-09-03

**Authors:** Jiaxin Xie, Benedict Tai, Tianchen Li, Lauren A. Lowe, Yi Shen

**Affiliations:** 1 School of Chemical and Biomolecular Engineering, The University of Sydney, Darlington NSW 2008, Australia; 2 Sydney Nano Institute, The University of Sydney, Camperdown NSW 2050, Australia

**Keywords:** biomolecular condensates, label–free techniques, liquid-liquid phase separation, liquid-to-solid transition, material properties

## Abstract

Biomolecular condensates are membrane-less compartments formed through phase separation that concentrate and organize biomolecules within cells. Far from being static droplets, they are dynamic molecular assemblies whose internal dynamics, structure, mechanics, and composition can evolve over time during cellular aging, disease-associated aggregation, or engineered material formation. These changes are often described using state descriptors such as liquid-like, gel-like, aged, or solid-like, yet similar apparent states can be interpreted differently depending on whether they are assessed in terms of morphology, dynamics, structure, mechanical response, or composition. The present review article summarizes an experimental framework used to probe biomolecular condensates and their transitions across complementary dimensions while minimizing perturbation to the system. A diverse suite of label-free optical, scattering, spectroscopic, mechanical, and microfluidic approaches is discussed, highlighting how integrated strategies can connect these readouts to enable more rigorous interpretations of condensate behavior. This framework is intended to guide researchers to move beyond broad material descriptors toward understanding the mechanisms underlying condensate evolution, the resolution of phase transitions, and their implications for biological function, pathology, and biomaterial design.

## Introduction

Biomolecular condensates create dynamic intracellular microenvironments that enrich specific proteins, nucleic acids, and other molecules to organize biochemical reactions, RNA metabolism, stress responses, signaling, and higher-order cellular architecture [1–3]. Condensates typically form through liquid–liquid phase separation (LLPS), but their properties are not fixed upon assembly: their composition, density, internal dynamics, viscoelastic response, interfacial behavior, and structural organization can evolve over time or in response to molecular and environmental cues [4–7]. This evolution can be diverted by adverse biological processes, such as cellular aging and mutation, leading to irreversible liquid-to-solid transitions (LSTs) contributing to pathological protein aggregation [8–11]. In neurodegenerative disease-associated systems, phase-separating proteins such as fused in sarcoma (FUS) can convert from dynamic liquid-like compartments into less reversible solid-like, hydrogel-like, or fibrillar assemblies, with disease-linked mutations accelerating this aberrant transition [8,12,13]. In engineered systems, in contrast, controlled condensate maturation or LSTs can be exploited to generate functional biomaterials [11,14]. Understanding condensate state transitions therefore extends beyond detecting phase separation and requires characterizing the molecular, structural, compositional, and mechanical changes that accompany condensate maturation and transformation [4,15].

Condensate states are often described using terms such as liquid-like, gel-like, aged, or solid-like, yet these descriptors can be misleading if they are treated as direct readouts of a single physical property [4,15,16]. A rounded droplet morphology, rapid molecular exchange, altered composition, or increased stiffness may each suggest aspects of condensate behavior, but none alone defines condensate state across all relevant length and time scales [4,5,17]. Instead, different techniques probe complementary aspects of condensate state across distinct spatial and temporal scales [5,18,19]. A central challenge is therefore to identify which aspect of the condensate state each observable directly reports and which aspects require further interpretation [4,18]. This challenge is compounded by the fact that common experimental tools are not always neutral observers [4,18]. Fluorescent tags, embedded tracer particles, applied forces, surface interactions, and imaging conditions can influence phase behavior, dynamics, or mechanical response, so the act of measurement may alter the system being measured [20–22]. Label-free and minimally perturbative approaches are therefore valuable because they can reduce measurement-induced distortion while providing access to complementary condensate-state readouts [18,19]. However, each method still has its own physical sensitivity, assumptions, resolution limits, and perturbative footprint [4,18].

Recent reviews have established important perspectives on condensate material properties, experimental best practices, label-free characterization, and microscopy-based analysis [4,18,19]. Building on these studies, we propose a minimally perturbative experimental readout framework that focuses on how complementary observables can be selected and integrated to resolve condensate state transitions. Rather than interpreting dynamics, structure, mechanics, and composition in isolation, we emphasize how these distinct but interrelated dimensions can be used together to determine when, where, and how condensate transitions emerge and evolve, while accounting for the perturbations and assumptions associated with each measurement. We therefore organize representative optical, scattering, spectroscopic, mechanical, and microfluidic approaches according to the experimental dimensions they constrain and highlight multimodal strategies that reduce ambiguity between non-equivalent readouts. Established methods are included where they provide important benchmarks or complementary information, while selected emerging approaches are discussed in greater detail when they offer capabilities particularly relevant to this framework. In this way, the framework is intended to move condensate characterization beyond single-technique state classification toward pathway-resolved and perturbation-aware interpretation.

## Experimental dimensions of condensate state

### Experimental dimensions that define condensate state

**Figure 1:**
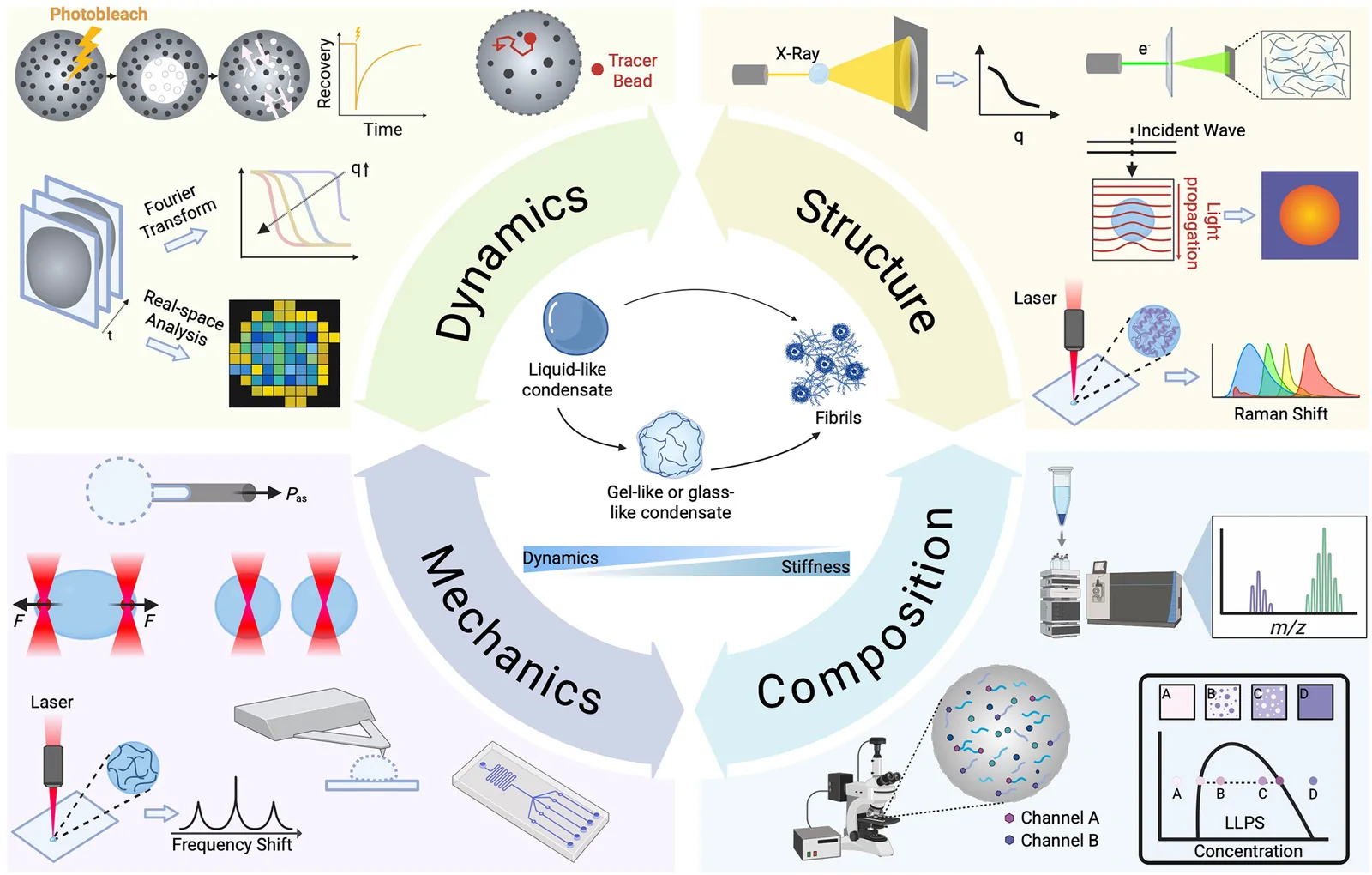
Experimental readout framework for interpreting biomolecular condensate state transitions Biomolecular condensates can evolve from dynamic liquid-like compartments toward gel-like, glass-like, or fibrillar/solid-like states. The surrounding framework groups representative approaches into four complementary readout classes: dynamics, structure, mechanics, and composition. These approaches report distinct observables, including molecular exchange and mobility, internal architecture, mechanical response, and molecular enrichment, and therefore constrain condensate state from complementary perspectives rather than providing a single definitive classifier. The circular arrangement highlights that these dimensions are mechanistically coupled but non-equivalent, and the corresponding experimental observables may evolve on different spatial and temporal scales during state transitions.

Condensates can span a broad range of material states and exhibit spatial and dynamic inhomogeneities whose significance depends on the spatial and temporal scales probed [4]. A more rigorous description of the condensate state therefore requires multiple experimental dimensions, including dynamics, structure, mechanics, and composition (Figure 1). These dimensions are mechanistically coupled but non-equivalent. For example, changes in condensate composition can alter intermolecular interactions and thereby influence molecular mobility, mechanical response, and structural organization, while dynamic arrest or structural maturation can in turn reshape local composition and partitioning. Importantly, changes across these dimensions do not necessarily occur synchronously and may emerge over distinct spatial and temporal scales during state transitions [9,10,23]. Therefore, no single measurement can fully define condensate state, and each readout instead constrains a particular aspect of the system within the limits and assumptions of the measurement [18,19]. This provides the basis for the integrative readout framework discussed below, in which the condensate state is interpreted through complementary experimental dimensions rather than through any single observable [18,22].

### What experimental observables can and cannot resolve

Having defined these dimensions, the next challenge is to interpret the observables used to access them. Similar experimental phenomena may be compatible with different underlying mechanisms, and different assays represent distinct aspects of condensate state [4,16]. Any inference about condensate identity, material state, or function is therefore limited by the physical sensitivity and conditions of the measurement, as well as by which alternative interpretations remain unresolved [15].

Although morphological readouts such as fusion, coarsening, and wetting are often used to interpret condensate behavior [17,24,25], similar morphologies do not necessarily imply identical internal states [26,27]. For example, fusion time is often treated as a viscosity proxy, yet this interpretation can fail in viscoelastic condensates, where finite shear-stress relaxation rather than simple viscocapillary flow can govern fusion dynamics [17]. Wetting poses a related caution, because morphology at an interface depends not only on condensate bulk properties but also on surface mechanics, adhesion, and interfacial energetics [25]. Morphology is therefore better treated as a mesoscale phenotype than as direct evidence for a specific material state or mechanism [26].

Dynamic measurements provide important information about condensate motion and exchange, but different readouts probe distinct physical processes [16]. Exchange-based readouts are most informative about molecules redistributed across condensate boundaries [28], whereas mobility-based readouts are more sensitive to local diffusion, confinement, and heterogeneity within the dense phase [29]. Deformation-based readouts probe how condensates respond over time to imposed deformations or mechanical stress [17], while fluctuation-based approaches capture the timescales of spontaneous local rearrangements across space [10,30]. Their interpretation should therefore be anchored to the dominant process being measured [16,28].

Structural readouts provide information about condensate organization, but the specific aspects they reveal depend strongly on the signal source and method [15]. These include internal architecture and connectivity [31,32], refractive-index-defined density variations [22,33], vibrational signatures of chemical environment or conformation [34,35], and ensemble-averaged structural organization across length scales [36,37]. Different structural measurements therefore constrain different aspects of condensate organization and are more informative when interpreted alongside dynamic, mechanical, or compositional readouts [15].

Compositional readouts identify which molecules are preferentially enriched or excluded from the condensed phase, making composition a distinct dimension of condensate organization and function [38]. Such information can be obtained through approaches ranging from mass spectrometry-based compositional profiling to partitioning measurements that quantify the enrichment of defined components. This selectivity is informative because partitioning reflects the interaction landscape and physicochemical environment of condensates rather than scaffold identity alone [39]. Large-scale partitioning studies have shown that small-molecule enrichment can vary by nearly six orders of magnitude across compounds, yet remain correlated across chemically distinct condensates [39]. Recent studies further extend compositional readouts beyond molecular inventory, showing that selective ion uptake and pH gradients can shape the chemical microenvironment inside condensates [40,41].

Mechanical readouts assess how condensates respond to force and deformation and therefore provide an important basis for distinguishing material regimes that are not readily resolved by other types of readouts [5,15]. Nonetheless, quantities such as viscosity, elasticity, or interfacial tension depend on the imposed perturbation, assay geometry, and the timescale over which the condensate response is measured [4,15]. This dependence is particularly clear for fusion-based and aspiration-based measurements, whose interpretation can shift when condensates depart from simple Newtonian behavior [17] or when analysis relies on specific hydrodynamic assumptions [42]. This limitation can be further amplified in aged or multiphase condensates, where region-specific relaxation can cause bulk mechanical values to obscure substantial spatial and dynamical heterogeneity within the condensate [10,15,43].

### Why minimally perturbative and integrated approaches are needed

These interpretive limits motivate two complementary experimental priorities: minimizing measurement-induced perturbation and integrating readouts that constrain different condensate-state dimensions. Experimental perturbations can arise from multiple aspects of the measurement configuration, including molecular labeling [21,22], optical exposure [4], substrate or interface interactions [44], sample confinement, and measurement-induced changes to the local environment. Among these factors, fluorescent tags have received particular attention because they are widely used in condensate studies yet have also been shown to alter phase separation behavior and compositional measurements [19,20,45]. For example, red fluorescent protein tagging has been shown to promote LLPS of huntingtin exon-1 under crowding conditions [21]. Label-free quantitative phase imaging (QPI) combined with analysis of tie-lines and refractive index (ATRI) further showed that fluorescent labels can alter condensate composition and compromise fluorescence-based concentration or partitioning measurements in multicomponent condensates containing FUS-related constructs [22].

These concerns are also relevant to widely used fluorescence-based readouts such as fluorescence recovery after photobleaching (FRAP), which probes molecular exchange by photobleaching fluorescently labeled molecules within a defined condensate region and monitoring fluorescence recovery over time. In addition to fluorescent labeling, the method involves localized photobleaching, while its interpretation can depend on condensate size and geometry, spatial heterogeneity, boundary effects, and model-dependent fitting of recovery curves [19,28]. FRAP therefore illustrates how a widely used readout can both provide valuable constraints on molecular exchange during condensate state transitions and involve multiple experimental factors that need to be considered in its interpretation [19,28]. Consequently, perturbation should be considered a property of the experimental configuration rather than of labeling status alone.

Robust condensate-state interpretation also requires integration across complementary readouts [18]. Integrated approaches combine observables that report on multiple dimensions of condensate organization, dynamics, composition, and mechanics [3,46]. This need is made especially acute in biomolecular condensates with internal heterogeneity and higher-order architecture, where different regions or scales may exhibit distinct behaviors that cannot be captured by any single readout alone [3,4].

Linking condensate state to biological outcome often requires controlled perturbations [45]. In this context, optogenetic and chemogenetic tools provide useful perturbation strategies, as they allow condensate assembly to be induced, reversed, or tuned with improved temporal and spatial control [47]. When paired with downstream biological output, such controlled perturbations can support more direct mechanistic interpretation [48]. Thus, label-free observation and controlled perturbation serve complementary purposes in linking condensate state to functional outcome: the former reduces measurement-induced distortion, whereas the latter introduces defined changes that can reveal causal relationships under controlled conditions. Integrated readouts are then needed to connect these perturbation responses to specific experimental dimensions [4].

To support method selection within this framework, Table 1 compares representative approaches in terms of their measurable parameters, spatial and temporal resolutions, throughput, perturbative footprint, and key limitations. Spatial and temporal resolution entries are representative and may depend on experimental implementation, whereas throughput and perturbative footprint are qualitative and context-dependent classifications. With this rationale in place, we next examine label-free optical and scattering approaches for probing condensate organization and dynamics.

**Table 1:** Comparative readout information, experimental characteristics, and perturbative footprint of representative methods for probing biomolecular condensates

Method	Information from readout	Spatial resolution	Temporal resolution	Throughput	Perturbation	Key limitation	Ref.
**Dynamics**							
**FRAP**	Molecular exchange and mobility	∼0.2–0.5 μm; diffraction-limited	ms–s	Moderate: sequential region of interest (ROI) measurements	Moderate–high: labeling and photobleaching	Recovery is geometry- and model-dependent	[19,28]
**Differential dynamic microscopy (DDM)**	Collective dynamics and viscoelastic behavior	*q*-dependent; no direct real-space resolution	ms–s	High: field-wide parallel analysis	Low: imaging; tracers optional	Limited spatial localization of intradroplet heterogeneity	[30,49,50]
**Spatial dynamic mapping (SDM)**	Spatially resolved dynamic heterogeneity	Sub-μm–μm; ROI dependent	ms–s	High: parallel ROI analysis	Low: imaging	Sensitive to acquisition and analysis parameters	[10]
**Single-molecule tracking**	Local molecular mobility and dynamic heterogeneity	∼10–50 nm	ms–s	Moderate: parallel trajectory acquisition	Moderate: fluorescent labeling and illumination	Sparse labeling and finite trajectory lengths can bias sampled dynamics	[51,52]
**Interferometric scattering correlation spectroscopy (iSCORS)**	Nanoscale condensation state and dynamics	Nanoscale-sensitive; diffraction-limited	μs–ms	High: wide-field parallel imaging	Low: coherent illumination	Background scattering complicates interpretation	[53]
**Structure**							
**Fourier-transform infrared spectroscopy (FTIR)**	Secondary structure and chemical environment	Several μm; wavelength-dependent	ms–s	Moderate: rapid spectral acquisition	Low: sample preparation	Limited spatial resolution in bulk measurements	[10,54]
**Raman microscopy**	Molecular composition and conformation	Sub-μm–μm	μs–s; modality- dependent	Low–moderate; modality-dependent	Low: laser exposure	Signal-speed trade off	[34,35,55,56]
**QPI**	Density, composition, and spatial organization	∼0.2–0.5 μm; diffraction-limited	ms–s	High: full-field parallel imaging	Low: transmitted illumination	Quantification requires optical and geometric assumptions	[22,57]
**Mechanics**							
**Brillouin microscopy**	High-frequency longitudinal mechanical response and dissipation	∼1 μm	ms–s	Moderate: pointwise spectral mapping	Low: optical exposure	Composition and hydration confound mechanical interpretation	[58–60,61]
**Atomic force microscopy (AFM)**	Local stiffness and elastic modulus	∼nm	ms–s	Low: serial pointwise mapping	High: contact and immobilization	Substrate and contact effects	[23,62]
**Micropipette aspiration (MPA)**	Surface tension and viscosity	Whole droplet	ms–s	Low: single-droplet measurements	High: aspiration and contact	Constitutive model assumptions can fail	[42,63]
**Optical tweezers**	Mechanical and interfacial relaxation behavior	∼μm	μs–ms	Low: single-object manipulation	High: optical force and heating	Mechanical inference is geometry- and model- dependent	[64,65,66]
**Passive microrheology**	Local viscosity and viscoelasticity	Sub-μm–μm	μs–ms	Moderate: simultaneous probe tracking	Moderate: embedded tracer	Probe-material interactions bias response	[67,68,69]
**Active microrheology**	Frequency-dependent viscous and elastic response	∼μm	μs–ms	Low: single-probe measurements	High: probe and applied force	Probe coupling can bias response	[70,71,72]
**Flicker spectroscopy**	Interfacial tension and bending rigidity	∼μm; diffraction-limited boundary imaging	ms–s	High: parallel boundary analysis	Low: imaging	Requires resolvable spontaneous boundary fluctuations	[73]
**Microfluidics**	Phase behavior and assembly response under controlled conditions	Detection-dependent	Detection- dependent	High: parallel droplet screening	Moderate: controlled environmental and mechanical perturbation	Device fabrication, operation, and standardization can be technically demanding	[74,75]
**Composition**							
**Mass spectrometry**	Molecular identity and abundance	No intrinsic spatial resolution	Endpoint; sampling- dependent	High: multiplexed molecular profiling	High: destructive sample isolation and processing	Native spatial organization and dynamics are lost during analysis	[76]
**Fluorescence-based partitioning analysis**	Partition coefficients and concentration ratios	Diffraction-limited	s–min	Moderate: multiple condensates per field	Moderate: fluorescent labeling commonly required	Partition coefficients depend on labeling, segmentation, and concentration calibration	[39]

## Label-free optical and scattering approaches for probing condensate organization and dynamics

The preceding section defines the condensate state in terms of the experimental dimensions that must be constrained. This section focuses on label-free optical, scattering, and spectroscopic approaches that primarily report on condensate density, dynamics, structure, and chemical composition. These techniques provide complementary ways to reveal how molecular interactions, cellular conditions, and disease-associated processes influence condensate organization and state across multiple experimental dimensions.

### Phase- and fluctuation-based imaging of condensate density and dynamics

**Figure 2:**
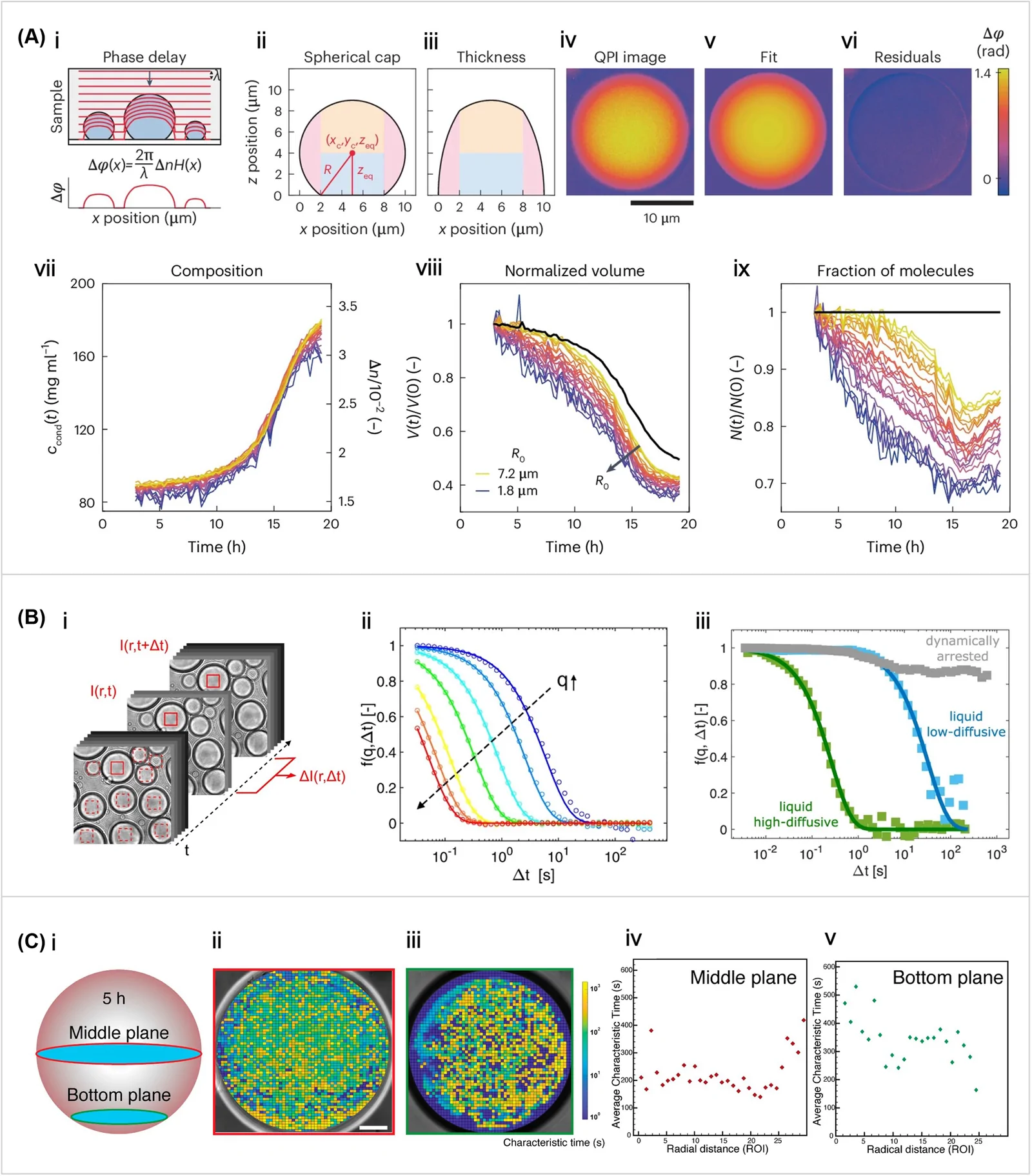
Phase- and fluctuation-based label-free imaging of condensate density and dynamics (**A**) QPI. (**i**) Schematic illustrating how condensates distort transmitted optical wavefronts and generate cumulative optical phase shifts. (**ii**) Geometric representation of a condensate approximated as a spherical cap. (**iii**) Corresponding local thickness profile used for reconstruction of condensate morphology and optical path length. (**iv**) Representative QPI phase image of a condensate. (**v**) Spherical-cap fit to the measured QPI image. (**vi**) Residual map showing agreement between the geometric model and the experimental phase profile. (**vii**) Time-dependent changes in condensate concentration and refractive-index contrast for condensates of different initial sizes from the same field of view. (**viii**) Normalized condensate volume over time, showing size-dependent shrinkage dynamics. (**ix**) Fraction of molecules remaining in individual condensates during aging; thick black lines indicate the expected trajectories if protein mass were conserved within the condensates. Adapted from ref [22] under a Creative Commons Attribution CC-BY International License. Copyright 2025 by the authors. (**B**) DDM. (**i**) Schematic of the DDM workflow, in which time-lapse brightfield images are converted into image-difference correlations and analyzed in Fourier space. (**ii**) Representative intermediate scattering functions, f(q, Δt), at different wavevectors, illustrating wavevector-dependent relaxation dynamics. (**iii**) Representative DDM readouts distinguishing liquid-like high-diffusive droplets, liquid-like low-diffusive droplets, and dynamically arrested condensates. Adapted from ref [30] under a Creative Commons Attribution CC-BY International License. Copyright 2022 by the authors. (**C**) SDM. (**i**) Schematic illustrating SDM measurements at different focal depths within an aged condensate. (**ii**,** iii**) Characteristic relaxation-time maps of a 5 h-aged condensate acquired at the middle plane and bottom plane, respectively, revealing depth-dependent dynamic heterogeneity. (**iv**, **v**) Radial distributions of the average characteristic relaxation time from the condensate center to the periphery for the middle and bottom planes, highlighting spatially nonuniform slowing within the same condensate. Adapted from ref [10] with permission from the authors. Copyright 2023 by the authors.

Brightfield and differential interference contrast microscopy have long served as label-free tools for visualizing biomolecular condensates, revealing characteristic droplet-like behaviors such as fusion, coarsening, and deformation [77]. While these methods capture droplet morphology and mesoscale behaviors, they provide limited quantitative insight into the optical-density changes that may underlie similar visual phenotypes. QPI (Figure 2Ai–vi) addresses this limitation by converting phase shifts in transmitted light into quantitative maps of optical path length, from which refractive-index or dry-mass distributions can be inferred. This enables real-time, label-free monitoring of condensate density and concentration-related changes [57]. Recent QPI studies of reconstituted condensates have directly tracked sequence-dependent density variations and complex aging dynamics in multicomponent condensates (Figure 2Avii–ix) [22]. When combined with ATRI, these measurements can further resolve multicomponent condensate composition in a label-free manner, including the concentrations of RNA and multiple RNA-binding-protein constructs, revealing that density and molecular composition can be decoupled [22]. The ability to distinguish density from composition is therefore important for understanding how changes in RNA or protein abundance reshape molecular partitioning within multicomponent condensates without necessarily altering their overall density.

DDM leverages intrinsic intensity fluctuations to extract droplet dynamics in an entirely label-free manner [49,50]. Originally developed for suspensions, DDM computes correlation functions from time-lapse microscopy images in Fourier space to probe wavevector-dependent dynamics in the sample (Figure 2Bi,ii) [49]. DDM can distinguish condensates spanning multiple dynamic states at the single-condensate level, including high-diffusive liquid-like droplets, low-diffusive liquid-like droplets, and dynamically arrested condensates (Figure 2Biii) [30]. For example, Linsenmeier et al. applied DDM to ATP/RNA-driven DEAD-box ATPase Dhh1 condensates and revealed that individual condensates within the same population exhibit distinct dynamic states, ranging from fast-diffusing liquid-like droplets to slowly relaxing viscous ones and nearly immobile, gel-like condensates [30]. By tracking how these dynamic states change with the ATPase activity of Dhh1, RNA structure, and aging, DDM enabled the effects of biochemical regulation to be resolved directly at the level of condensate material state [30].

SDM was introduced to resolve spatiotemporal variations of condensate dynamics within individual droplets [10]. SDM analyzes local intensity fluctuations across an individual condensate and maps the characteristic relaxation time at each ROI, thereby revealing spatial heterogeneity in internal dynamics. By adjusting the depth of field, SDM can focus selectively on different *z*-planes within a condensate and map the dynamics accordingly (Figure 2Ci). Using this approach, Shen et al. demonstrated nonuniform solidification in aging FUS condensates, revealing coexistence of more liquid-like and more arrested regions within the same droplet, with stiff, arrested regions nucleating at the condensate periphery (Figure 2Cii–v) [10]. Such dynamic heterogeneity is now frequently observed during condensate maturation: as time progresses or under external stimuli, some regions within condensates remain fluid while others progressively solidify [10,30,78]. Accordingly, label-free fluctuation-based techniques, including DDM, SDM, and related dynamic speckle analyses, are emerging as useful tools for detecting spatiotemporal heterogeneity and the onset of dynamical slowing or arrest in biomolecular condensates [30,79]. Such dynamical slowing has been reported before obvious fibrillar or solid-like structures become apparent [80]. Resolving when and where dynamic arrest first develops can therefore provide insight into the progression of condensate maturation, with potential implications for pathological aggregation.

### Brillouin light scattering for spatially resolved mechanical mapping

**Figure 3:**
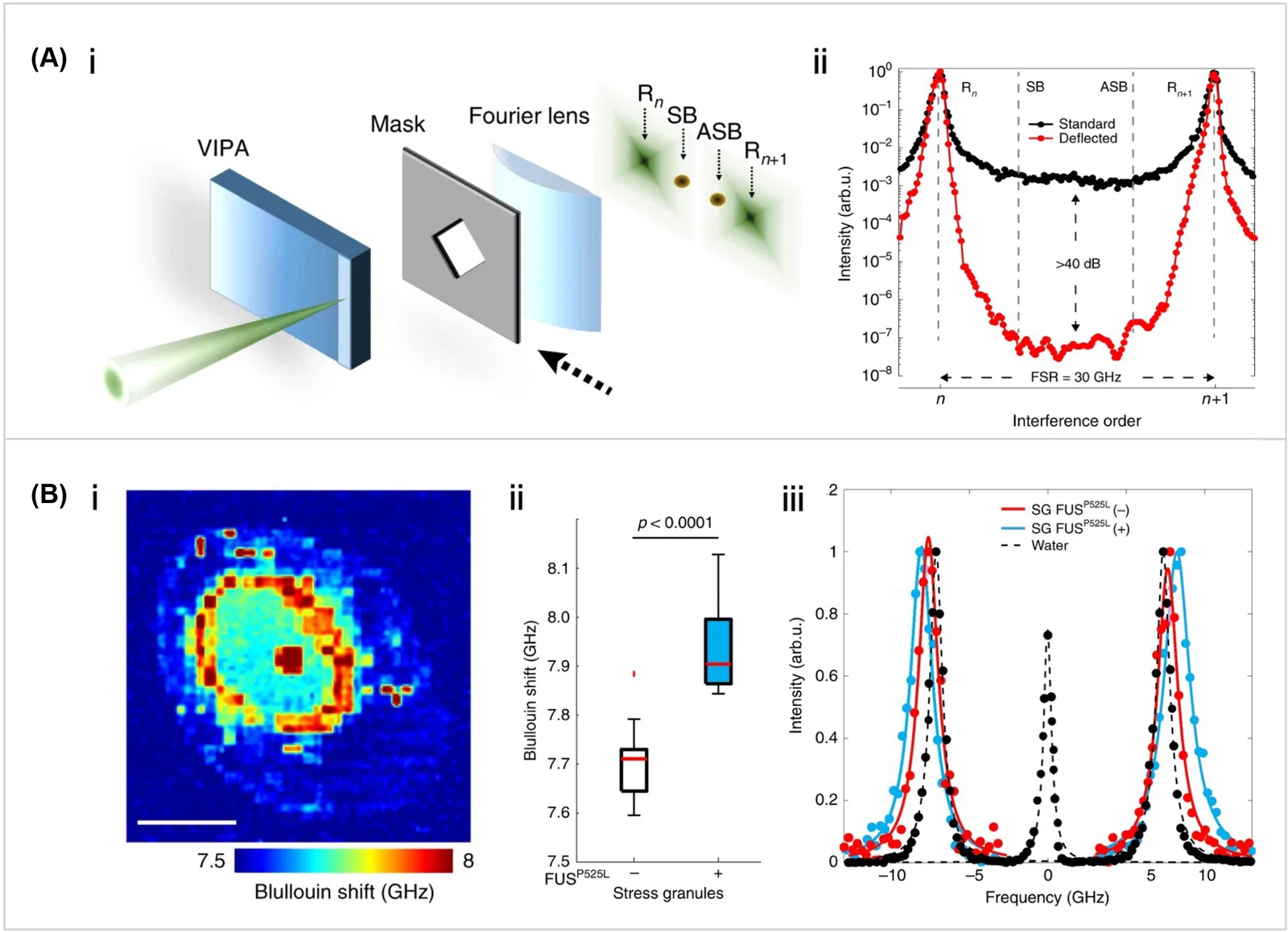
BLS microscopy for label-free mechanical mapping of condensates (**A**) Brillouin spectral readout and instrumental optimization. (**i**) Schematic of a background-deflection virtually imaged phased array spectrometer with a rhomboidal mask for suppressing elastic background and enhancing Brillouin sideband visibility. (**ii**) Spectral intensity profiles showing improved Rayleigh-background rejection in the deflected configuration. (**B**) BLS analysis of stress granules (SGs) associated with mutant FUS expression. (**i**) Representative Brillouin shift map of a stressed cell expressing mutant FUS. (**ii**) Quantification of Brillouin shifts showing elevated values in SGs relative to the surrounding cellular background. (**iii**) Representative Brillouin spectra of water and SGs with or without mutant FUS expression, showing both increased frequency shift and broader linewidth in the mutant FUS condition. Figure 3A,B were adapted from ref [58] under a Creative Commons Attribution CC-BY International License. Copyright 2018 by the authors.

Brillouin light scattering (BLS) microscopy exploits gigahertz-frequency inelastic scattering of light by thermally excited acoustic phonons to probe high-frequency viscoelastic properties (Figure 3Ai, ii) [59]. In this readout, the Brillouin frequency shift is linked to the sample’s longitudinal elastic modulus, whereas the Brillouin linewidth reflects viscous damping [59,60]. As a purely optical and label-free technique, BLS enables non-contact three-dimensional mechanical mapping of live cells and other soft biomaterials [59,60,81]. Its application has expanded substantially in mechanobiology, supported by confocal implementations and improved spectrometer designs that have enhanced spectral resolution and imaging speed [58,82,83]. These advances position BLS as an attractive approach for probing biomolecular condensates, particularly where traditional physical probes are difficult to apply or may perturb the material being measured [19,84].

Applications of BLS in condensate studies have shown its ability to resolve mechanical contrasts between condensate states. For example, background-deflection BLS imaging revealed that arsenite-induced SGs in cells expressing ALS-associated FUS^P525L^ exhibited higher Brillouin shifts than SGs formed without mutant FUS expression, together with broader Brillouin linewidths, consistent with a more solidified and viscously damped material state during pathological assembly (Figure 3Bi–iii) [58]. Combined fluorescence and Brillouin mapping in Ras-GTPase-activating protein-binding protein 1 (G3BP1)-green fluorescent protein (GFP)-expressing cells further showed condition-dependent mechanical differences in stress-induced condensates, with the largest Brillouin shifts observed when arsenite stress was coupled to FUS^P525L^ expression, indicating that disease-associated perturbations can drive SGs toward a mechanically more solid-like state [84]. More broadly, the ability of BLS to resolve pathological mechanical contrast extends beyond intracellular SGs. In *ex vivo* Alzheimer’s disease tissue, micro-Brillouin mapping combined with hyperspectral or correlative Raman analysis has shown that amyloid-β plaques exhibit elevated Brillouin shifts relative to adjacent tissue and pronounced internal mechanical heterogeneity, consistent with a stiff core surrounded by a more viscoelastic peripheral layer [85,86]. Together, these studies show that stress- and disease-associated condensate reorganization can involve distinct changes in mechanical properties.

Despite its promise, BLS data must be interpreted carefully. The measured Brillouin shift can be influenced not only by intrinsic elastic properties but also by factors like molecular composition and water content, so rigorous calibration and appropriate controls are essential [61]. Recent comprehensive reviews provide guidelines on best practices and describe technical improvements, ranging from optimized spectrometer designs to advanced background suppression and computational analysis, that enhance sensitivity while minimizing photodamage [59,60]. BLS imaging remains constrained by diffraction-limited spatial resolution and a relatively slow acquisition speed, and it requires high-irradiance illumination that can stress delicate samples. Moreover, widespread adoption is limited by the need for custom-built instrumentation [18]. Nonetheless, ongoing developments such as artificial intelligence (AI)-assisted data processing and nascent superresolution BLS approaches offer promising avenues to overcome these challenges, further expanding the applicability of Brillouin microscopy to condensate biology and related soft-matter systems [18,59,60].

### Vibrational spectroscopy of condensate chemical and structural heterogeneity

Vibrational spectroscopy provides label-free access to condensate chemistry and molecular structure by probing molecular vibrations [34,56]. Infrared approaches such as FTIR spectroscopy are well suited to ensemble-level analysis of protein secondary structure and hydration state [23,54], whereas Raman-based approaches can provide chemically specific readouts of composition and structure, with imaging implementations enabling spatial mapping in selected condensate systems [34,56].

**Figure 4:**
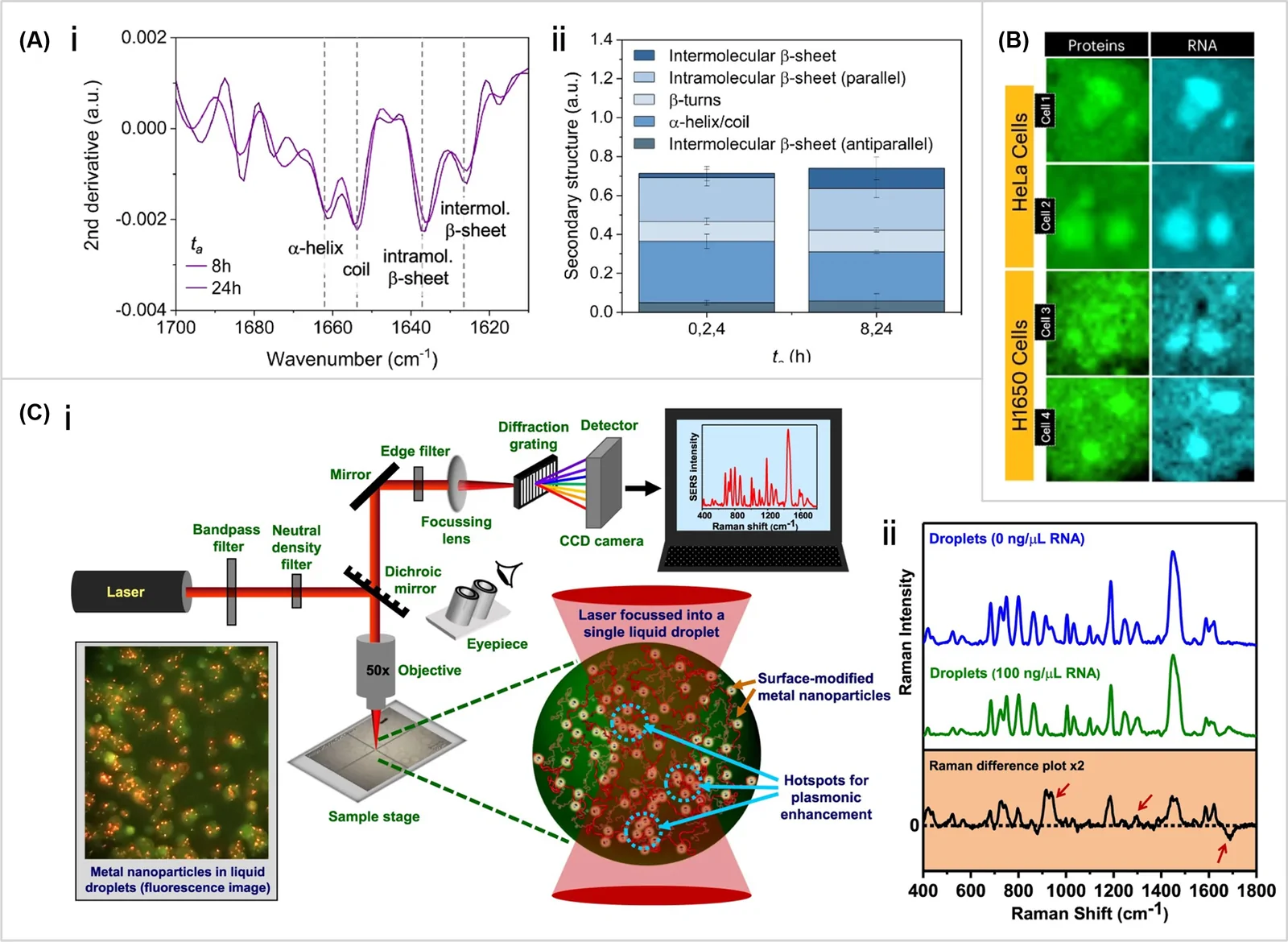
Vibrational-spectroscopy-based readouts of condensate chemical and structural heterogeneity (**A**) FTIR analysis of aging FUS condensates. (**i**) Second-derivative amide-I-region spectra showing aging-associated changes in secondary-structure signatures. (**ii**) Quantification of secondary-structure contributions, including β-sheet-associated components, at different aging times. Adapted from ref [23] under a Creative Commons Attribution CC-BY International License. Copyright 2025 by the authors. (**B**) Label-free Raman microspectroscopy maps protein- and RNA-rich intracellular condensate-like regions using intrinsic vibrational contrast. Adapted from ref [55] under a Creative Commons Attribution CC-BY International License. Copyright 2022 by the authors. (**C**) Single-droplet surface-enhanced Raman spectroscopy (SERS) analysis of FUS condensates. (**i**) Schematic of the SERS platform used to analyze FUS condensates containing surface-functionalized silver nanoparticles. (**ii**) Representative single-droplet SERS spectra of FUS condensates in the absence or presence of RNA, together with a Raman difference spectrum highlighting RNA-dependent spectral changes. Adapted from ref [35] under a Creative Commons Attribution CC-BY International License. Copyright 2022 by the authors.

FTIR has been especially informative for identifying structural transitions during condensate maturation or LSTs, revealing increasing β-sheet-associated signatures during aging (Figure 4Ai,ii) [10,23]. In shear-mediated condensate aging, FTIR measurements supported the interpretation that flow-induced LSTs involve structural conversion from disordered protein states toward β-sheet-rich assemblies [74]. More recently, FTIR analysis of aging FUS condensates first assessed condensate-associated hydration after deposition and then tracked changes in the amide-I region and hydrogen–deuterium exchange associated with secondary-structure reorganization, revealing progressive water loss and the emergence of intermolecular β-sheet-rich signatures during aging [23]. Emerging infrared vibrational approaches, including two-dimensional infrared spectroscopy, can resolve interaction-dependent secondary-structure formation and ultrafast hydrogen-bond or hydration dynamics in condensates, thereby extending infrared readouts from overall water content and structural fingerprints toward local solvation and interaction dynamics [87–89]. These measurements connect condensate aging to the molecular events underlying pathological conversion by revealing whether loss of liquid-like behavior coincides with the emergence of intermolecular β-sheet interactions characteristic of aggregation-prone states.

Raman-based methods provide complementary vibrational information and have greater potential for spatially resolved chemical mapping [90,91]. Label-free Raman microspectroscopy has been used to distinguish intracellular condensate-like regions, including nucleoli and Cajal bodies, from the surrounding nucleoplasm using intrinsic protein- and RNA-associated vibrational contrast (Figure 4B) [55]. The ability to distinguish nucleoli and Cajal bodies through their intrinsic molecular signatures enables the biochemical organization of nuclear condensates to be tracked across cellular states, including their remodeling during mitosis [55]. Spontaneous Raman mapping has also been used to quantify macromolecular enrichment within phase-separated compartments using the Raman bands of water as an internal intensity standard, enabling *in situ* estimates of protein concentration and molecular crowding in both reconstituted droplets and in live cells [92]. Beyond spontaneous Raman, condensate studies also benefit from coherent Raman techniques, such as stimulated Raman scattering (SRS) [34,93] and broadband coherent anti-Stokes Raman scattering (BCARS) [94,95], as well as signal-enhanced implementations such as SERS [96,97], which improve sensitivity and speed for condensate analyses. For example, a single-droplet SERS platform resolved conformational heterogeneity and specific protein–RNA interactions within individual FUS condensates, enabling compositional and structural analysis at single-condensate resolution (Figure 4Ci) [35]. Using this approach, RNA binding was found to partially unwind the FUS RNA-binding domain, as indicated by RNA-dependent shifts in secondary-structure-associated Raman bands (Figure 4Cii) [35]. For disease-associated proteins, spatially resolved secondary-structure imaging can reveal how sequence variation and protein-RNA interactions shape the structural evolution of aging condensates toward aggregation-prone β-sheet-rich states [35]. In addition, coherent Raman approaches have been used to monitor LLPS and its subsequent structural evolution with chemical specificity, underscoring the value of Raman-based methods for time-resolved studies in which phase transitions are accompanied by compositional or conformational changes [94,95,98].

### Interferometric scattering for nanoscopic condensate mass and motion

Interferometric scattering (iSCAT) microscopy exploits the interference of light scattered by nanoscale objects with a reference beam to detect extremely small masses and motions [99,100]. The detected intensity contains a cross-term between the scattered field and reference field that amplifies the weak Rayleigh scattering signal [99,100]. This interferometric amplification enables label-free detection of individual biomolecules well below the diffraction limit, including individual proteins in solution [101].

**Figure 5:**
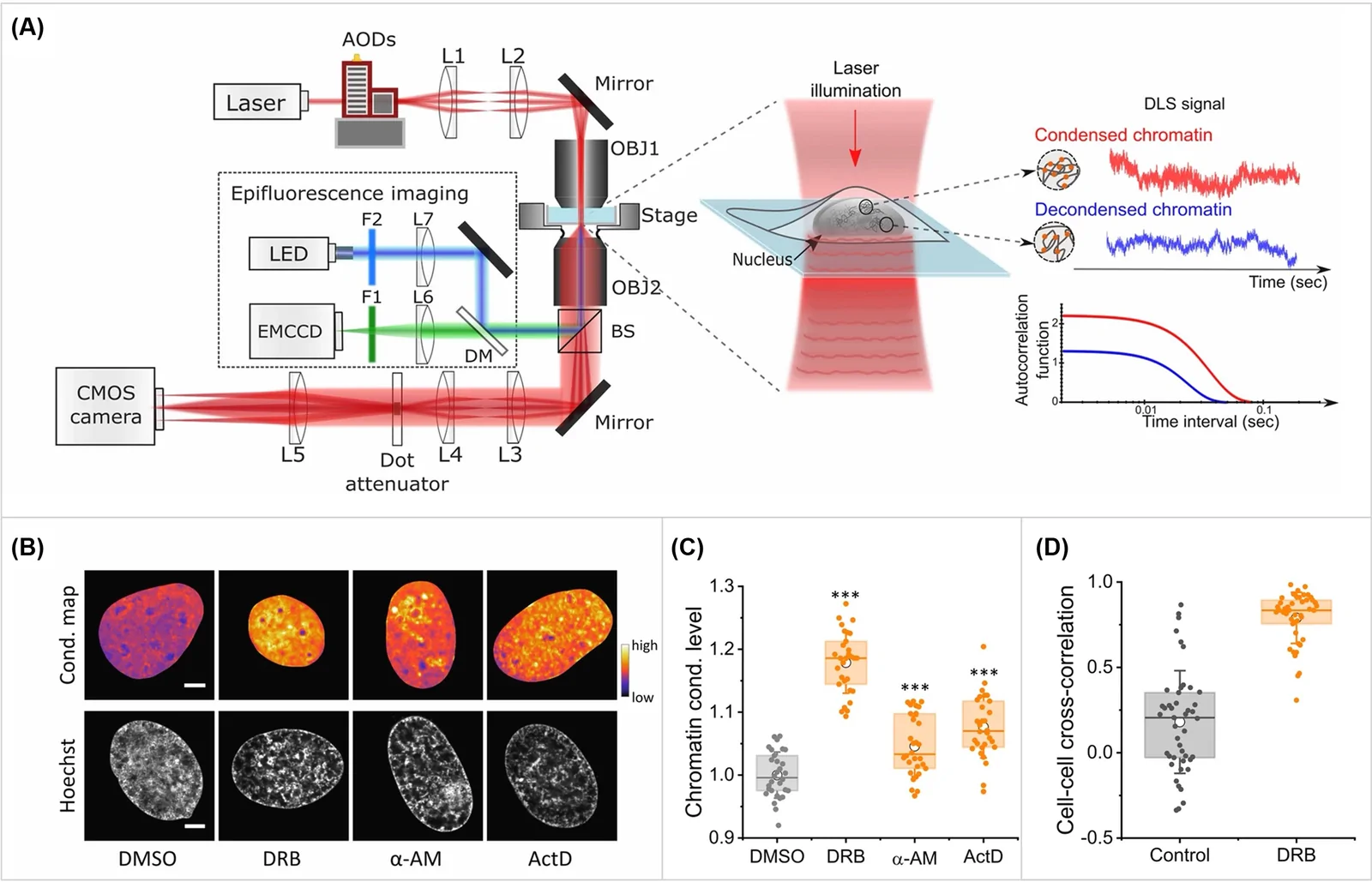
iSCORS for label-free mapping of chromatin condensation dynamics (**A**) Schematic of iSCORS microscopy, in which dynamic scattering signals are recorded and analyzed to infer chromatin condensation states and dynamics. (**B**) Representative chromatin condensation maps and corresponding Hoechst images of nuclei under control dimethyl sulfoxide (DMSO) and transcription-inhibiting treatments, including DRB, α-amanitin (α-AM), and actinomycin D (ActD). Scale bars represent 5 μm. (**C**) Quantification of chromatin condensation levels under different transcription-inhibition conditions. (**D**) Cell-cell cross-correlation of chromatin condensation dynamics, showing increased synchrony in DRB-treated cells. Figure 5A–D were adapted from ref [53] under a Creative Commons Attribution CC-BY International License. Copyright 2024 by the authors.

One powerful extension of iSCAT is iSCORS, which leverages temporal intensity fluctuations to map nanoscale dynamics (Figure 5A). iSCORS has been applied in live-cell nuclei to probe chromatin compaction and chromatin-compartment coupling. By analyzing the scattering fluctuations from moving nucleosomes, iSCORS can spatially resolve chromatin condensation states without requiring DNA labels, generating maps of chromatin nano-organization based on scattering variance and apparent diffusion coefficients (Figure 5B) [53]. These readouts distinguish more condensed chromatin from surrounding nuclear regions and reveal perturbation-dependent changes in chromatin organization that are difficult to discern by diffraction-limited DNA staining alone [53]. Upon transcription inhibition, iSCORS reveals treatment-dependent nanoscale reorganization of chromatin, manifested as increased chromatin condensation and enhanced coordination of condensation dynamics across cells, particularly under 5,6-dichloro-1-β-D-ribofuranosylbenzimidazole (DRB) treatment (Figure 5C,D) [53]. These observations connect nanoscale chromatin dynamics to transcriptional state, illustrating how changes in nuclear function can be accompanied by spatial reorganization that remains difficult to resolve from conventional nuclear morphology alone [53,102].

Viewed across these approaches, different aspects of condensate maturation may become detectable at different stages, while compositional or conformational changes may occur without an equivalent change in morphology [10,22,23,34]. This makes the relationship between readouts informative in itself: concordant changes strengthen the assignment of a broader state transition, whereas their decoupling can reveal intermediate stages that would be obscured by an endpoint descriptor such as ‘liquid-like’ or ‘solid-like.’ Mechanical measurements discussed in the following section provide an additional experimental dimension by determining whether these molecular and dynamic changes are accompanied by altered deformation, viscoelasticity, stiffness, or interfacial response.

## Probing condensate material properties with mechanical readouts

Mechanical readouts assess condensate material properties within defined experimental geometries and timescales. These approaches include aspiration, tracer-based assays, indentation, optical manipulation, and microfluidic assays, which can be used to probe bulk rheology, local viscoelasticity, stiffness, and interfacial mechanics. The material parameters measured should be interpreted as assay-dependent constraints rather than intrinsic state labels. Tracking these mechanical responses across changes in composition, maturation, or environmental conditions can reveal how condensate material properties evolve alongside the molecular and cellular processes that regulate them.

### Direct mechanical interrogation of condensates

Direct mechanical interrogation applies controlled force or deformation to individual condensates and is therefore useful when condensate mechanics need to be measured at the single-droplet level, especially during aging or disease-associated transitions. These methods test whether condensates behave mechanically as simple viscous liquids, viscoelastic fluids, or more arrested materials under defined perturbation, helping to interpret changes in liquid-like behavior, elasticity, or interfacial mechanics in biologically and pathologically relevant systems [4,5].

**Figure 6:**
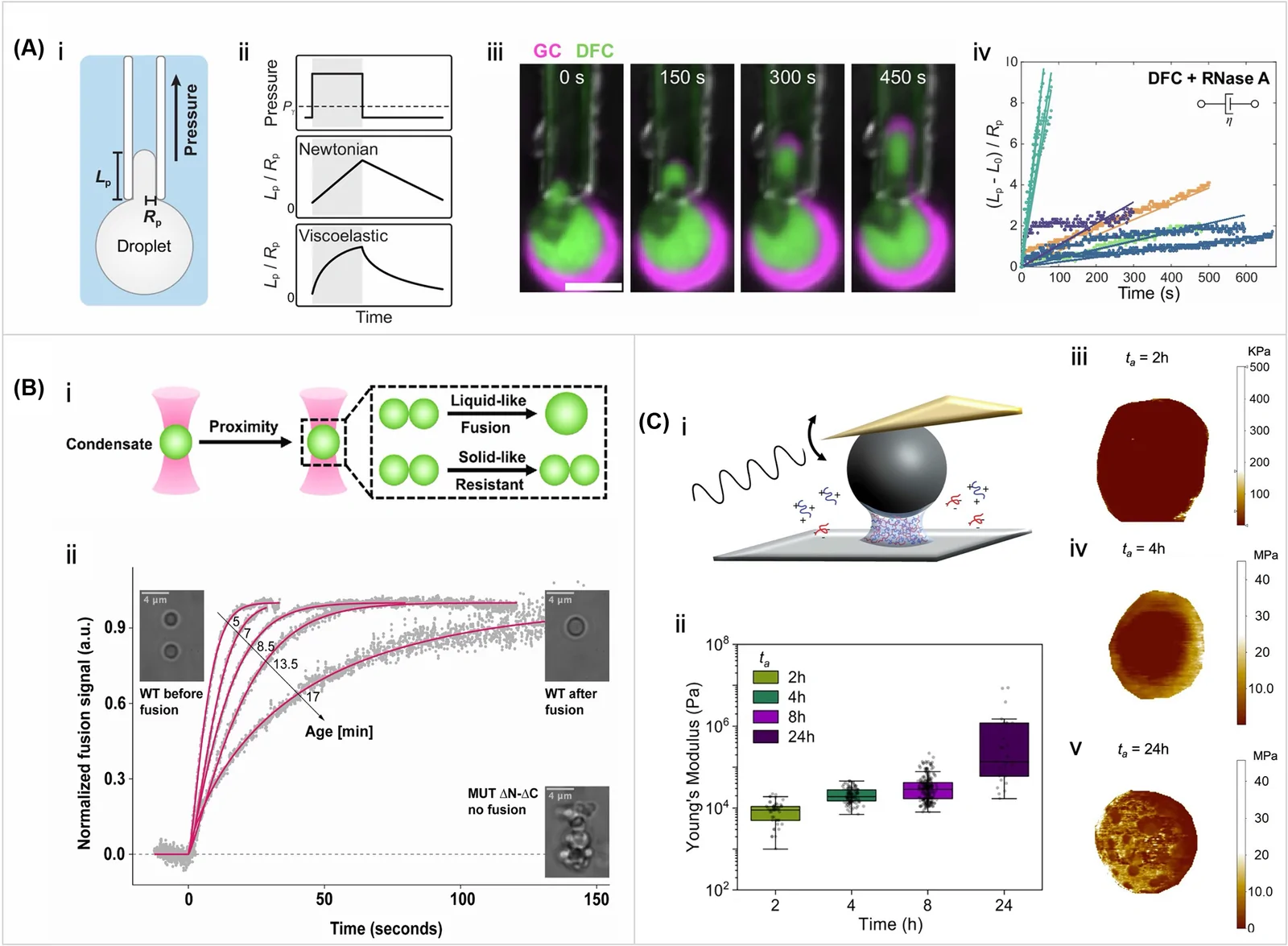
Direct mechanical interrogation of condensate material properties (**A**) MPA. (**i**) Schematic of aspiration of a nonwetting droplet into a micropipette. (**ii**) Idealized strain responses of Newtonian and viscoelastic fluids under applied and released pressure. (**iii**) Representative aspiration of an RNase-treated nucleolus, showing the granular component (GC) and DFC. (**iv**) Aspiration responses of RNase-treated DFCs, showing more linear, Newtonian-like behavior after RNase treatment. Adapted from ref [103] under a Creative Commons Attribution CC-BY International License. Copyright 2025 by the authors. (**B**) Optical tweezers. (**i**) Schematic of a dual-trap fusion assay used to classify condensates as liquid-like, fusion-defective, or resistant to fusion. Adapted from ref [104] under a Creative Commons Attribution CC-BY International License. Copyright 2023 by the authors. (**ii**) Fusion kinetics of purified GFP-Me31B (pMe31B) condensates at different aging times, with the ΔN-ΔC mutant showing little or no productive fusion. Adapted from ref [64] under a Creative Commons Attribution CC-BY International License. Copyright 2021 by the authors. (**C**) AFM. (**i**) Schematic of an AFM-based nanomechanical assay using a colloidal probe to interrogate condensates on a substrate. Adapted from ref [62] under a Creative Commons Attribution CC-BY International License. Copyright 2023 by the authors. (**ii**) Young’s modulus of single FUS condensates as a function of aging time. (**iii–v**) Representative AFM nanomechanical maps of individual FUS condensates showing progressive stiffening and mechanical heterogeneity during aging. Panels (Cii–v) were adapted from ref [23] under a Creative Commons Attribution CC-BY International License. Copyright 2025 by the authors.

MPA has been widely adopted to quantify condensate surface tension and viscosity (Figure 6Ai,ii) [63]. In one demonstration, aspiration of LAF-1 RGG protein condensates provided direct measurements consistent with fusion and FRAP assays [63]. More recently, a calibration-free hydrodynamic model has improved the extraction of condensate properties from aspiration data and reduced dependence on empirical calibration [42]. However, standard MPA analyses assume homogeneous Newtonian liquid behavior, which may not hold for all biomolecular condensates. Wang et al. [63] note that many condensates exhibit viscoelastic or gel-like responses, showing an immediate elastic deformation followed by slow creep under a step pressure, phenomena that linear Newtonian fits cannot capture [4,42,63]. Consequently, MPA can underrepresent the elastic component of droplets that deviate from simple liquids. Nonetheless, MPA remains valuable for comparing relative mechanics: aspiration measurements on *Xenopus* nucleoli revealed RNA-dependent differences in viscoelasticity and interfacial tension across nucleolar subcompartments, and representative aspiration responses showed that RNase treatment shifted dense fibrillar component (DFC) behavior toward a more linear, Newtonian-like response (Figure 6Aiii,iv) [103]. This RNA-dependent mechanical response provides a physical link between nucleolar composition and material state, showing how changes in a functional molecular component can reorganize the mechanics of nucleolar subcompartments.

Optical tweezers provide another route to direct mechanical interrogation, with the advantage of highly controlled force application during droplet deformation and fusion assays [65]. Optical tweezers can be used to manipulate condensate interfaces, control droplet coalescence, and assess how condensates relax after an imposed deformation (Figure 6Bi) [65,66,70,71]. Using a dual-trap optical tweezers setup, Ghosh and Zhou showed that the fusion speeds of different biomolecular droplets span two orders of magnitude, indicating that condensates with similar droplet-like appearance can nevertheless differ substantially in force-response behavior [66]. Dual-trap optical tweezers have also been used to quantify age-dependent fusion in purified GFP-Me31B (pMe31B) condensates, revealing progressively slower fusion dynamics as condensates mature, whereas the ΔN-ΔC mutant showed little or no productive fusion (Figure 6Bii) [64]. These age-dependent changes in fusion behavior are also linked to P-body function during development, as the physical state of Me31B condensates regulates the storage and release of bicoid mRNA [64]. However, optical tweezer measurements are not perturbation-free: local heating, probe geometry, and the relation between the trapped object and the condensate network all influence interpretation [4,65,71].

AFM provides a complementary contact-based route to higher-resolution mechanical characterization, particularly for condensates that are surface-associated, mechanically heterogeneous, or no longer well described as simple liquids. Colloidal probe AFM with small-amplitude oscillatory indentations has enabled frequency-resolved microrheology on protein droplets (Figure 6Ci) [62]. For instance, Li et al. [62] accessed oscillation frequencies of 1–10 rad s^−1^, revealing three mechanical regimes: an interfacial tension-dominated response at low frequencies, a viscous-to-elastic crossover at intermediate timescales, and a predominantly elastic behavior at higher frequencies. Advanced AFM approaches also enable nanoscale stiffness mapping: AFM-based nanomechanical measurements on aging FUS condensates showed that Young’s modulus increased over time, while stiffness maps revealed progressively more heterogeneous and solid-like behavior (Figure 6Cii–v) [23]. Such nanoscale mechanical heterogeneity can reveal intermediate states along pathological maturation pathways, including local stiffening that may precede complete macroscopic solidification and viscoelastic changes that are not captured by single-timescale measurements [23]. PeakForce Quantitative Nano-Mechanics (PF-QNM) further allows nanoscale stiffness mapping [105]. Chuang et al. [106] used PF-QNM to monitor a light-triggered LST in a peptide/FUS condensate system, finding that the apparent Young’s modulus increased from ∼87 MPa in the liquid droplet to ∼439 MPa after conversion to amyloid-like fibrils. Naghilou et al. demonstrated that an AFM-based scanning probe assay can characterize the gelation of polyelectrolyte condensate droplets and their mechanical ‘rejuvenation’ after chemical treatment. By leveraging rapid high-force indentations and concurrent nanoscale imaging, this approach overcomes the small-force limitations of traditional rheology [107]. The trade-off, however, is that AFM is inherently contact-based and often requires sufficient substrate adhesion for stable interrogation. This constraint should be considered when applying scanning-probe methods to highly dynamic droplets [108].

### Probe-based and fluctuation-based rheological readouts

Probe-based and fluctuation-based approaches infer condensate material properties from local tracer motion or spontaneous interfacial fluctuations, rather than from direct whole-droplet deformation [4,68]. By sampling local motion or boundary dynamics, these methods are useful for small condensates, mechanically heterogeneous systems, and droplets whose properties evolve over time [5,68].

**Figure 7:**
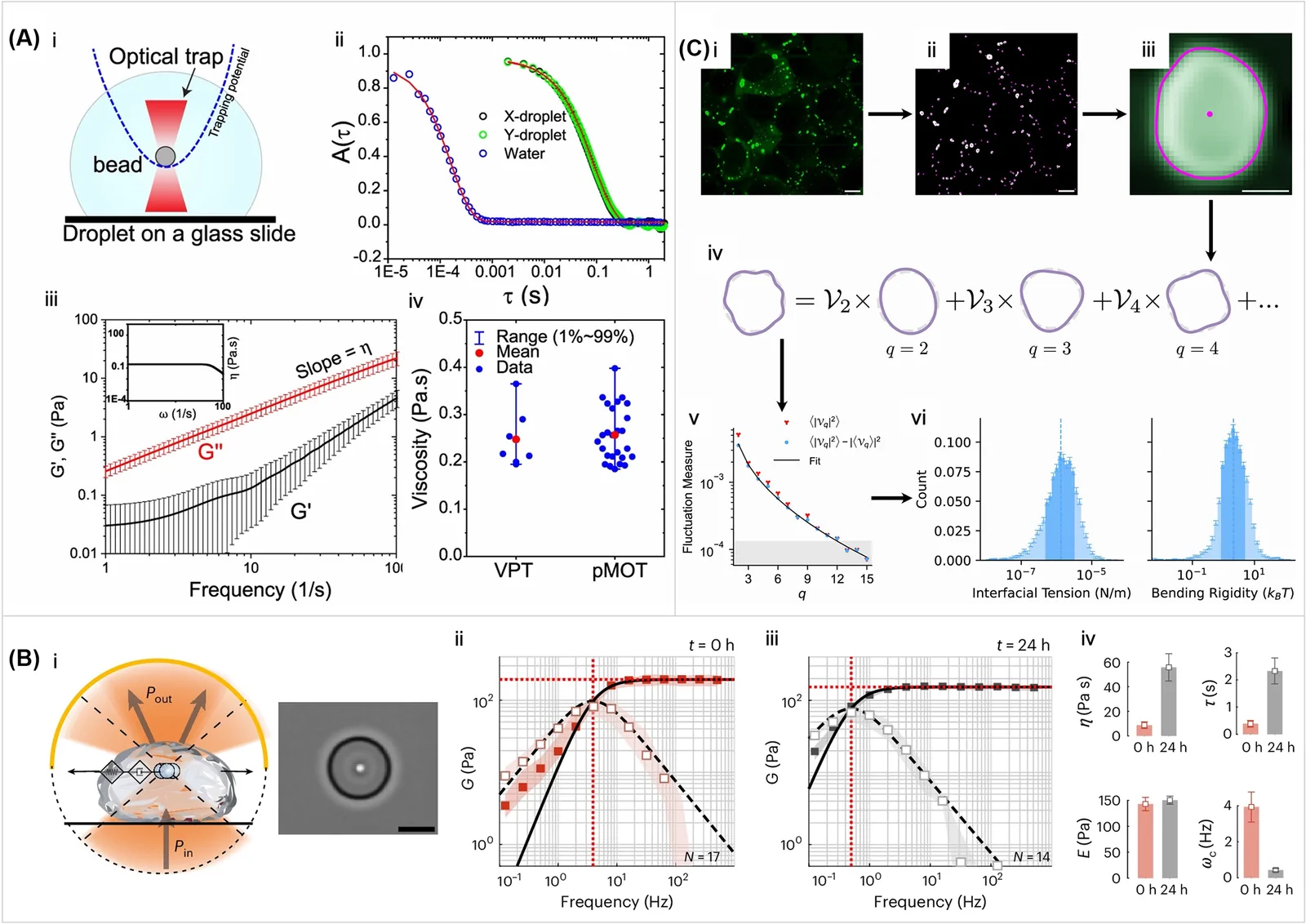
Probe-based and fluctuation-based readouts of condensate mechanics (**A**) Passive microrheology of [KGKGG]_5_–rU40 protein–RNA condensates formed from lysine-rich repeat polypeptide [KGKGG]_5_ and homopolymeric RNA rU40. (**i**) Schematic of passive optical-trap microrheology with a bead optically trapped inside a condensate. (**ii**) Normalized position autocorrelation function for trapped beads in condensates along the *x*-direction (black) and *y*-direction (green), with water shown as a reference (blue). (**iii**) Frequency-dependent viscoelastic moduli derived from normalized position autocorrelation functions, where G′ and G″ represent the elastic and viscous moduli, respectively. (**iv**) Comparison of zero-shear viscosity obtained from VPT and passive optical-trap microrheology (pMOT). Adapted from ref [67] under a Creative Commons Attribution CC-BY International License. Copyright 2021 by the authors. (**B**) Active microrheology of protein droplets formed by the *C. elegans* proteins MEC-2 and UNC-89. (**i**) Schematic of time-shared optical tweezer microrheology (TimSOM) on a condensate with an embedded bead. (**ii**,** iii**) Storage (G′, filled circles) and loss (G″, open circles) moduli measured at 0 h and 24 h after condensate formation. (**iv**) Age-dependent changes in fitted rheological parameters, including viscosity (*η*), stiffness (*E*), relaxation time (*τ*), and crossover frequency (*ω_c_*). Adapted from ref [72] under a Creative Commons Attribution CC-BY International License. Copyright 2025 by the authors. (**C**) Interfacial fluctuation analysis of SG condensates by flicker spectroscopy. (**i–iii**) Workflow for identifying condensates, locating their centers, and extracting their boundaries. (**iv**) Fourier decomposition of condensate boundary fluctuations. (**v**) Fitting of the fluctuation power spectrum to infer interfacial tension and bending rigidity. (**vi**) Population distributions of interfacial tension and bending rigidity obtained from many condensates. Adapted from ref [73] under a Creative Commons Attribution CC-BY International License. Copyright 2025 by the authors.

Passive microrheology, most commonly implemented through video particle tracking (VPT), is widely used to estimate condensate viscosity by following the Brownian motion of embedded tracer beads [68]. From the mean-squared displacement of these probes, an apparent viscosity can be inferred using the Stokes–Einstein relation, provided that bead motion reflects local condensate rheology rather than probe-specific artifacts such as edge interactions or bead-network adhesion [68]. Combining particle-tracking and fusion measurements can, in principle, enable inference of both viscosity and surface tension. However, when condensates exhibit viscoelastic behavior, shear-stress relaxation can delay droplet fusion beyond predictions of a simple Newtonian viscocapillary model [17]. Passive particle tracking can also report local rheological heterogeneity when different regions of a condensate exhibit distinct probe dynamics [69]. Optical trap-based passive microrheology extends this approach by analyzing fluctuations of a trapped bead, enabling extraction of autocorrelation functions, frequency-dependent viscoelastic moduli, and zero-shear viscosity (Figure 7Ai–iv) [67]. Because passive microrheology does not require fluorescence labeling of the biomolecules under study, it can provide viscosity information without directly modifying the condensate-forming macromolecules, while revealing how changes in intermolecular interaction networks reshape the local material environment experienced by molecules within the dense phase [68]. However, the method is not probe-neutral: bead size, surface chemistry, passivation quality, and interactions between probes and condensate components can all affect the measured response [19,68]. It also becomes impractical when condensates are too small relative to probe size or when probes sample boundaries rather than condensate interiors [68].

Active microrheology builds on probe-based rheological measurements by applying a controlled force to an embedded probe, commonly through optical or magnetic tweezers, and measuring the resulting displacement or strain response [70,71]. Compared with passive particle tracking, active approaches enable more direct interrogation of viscoelastic behavior because oscillatory forcing enables estimation of frequency-dependent storage and loss moduli across multiple timescales [70,71]. Català-Castro et al. developed time-shared optical tweezer microrheology, which uses two near-instantaneous optical traps on a single embedded bead to measure frequency-dependent viscoelasticity and resolve age-dependent changes in *Caenorhabditis elegans* MEC-2/UNC-89 protein droplets (Figure 7Bi–iv) [72]. Resolving this frequency dependence reveals that the apparent mechanical response of a condensate can vary across timescales, transitioning from predominantly fluid-like to increasingly elastic behavior, thereby influencing molecular diffusion, deformation, and internal rearrangement [62,70,71]. Peddireddy et al. introduced optical-tweezers-integrating-differential-dynamic-microscopy (OpTiDDM), which combines localized optical forcing with fluctuation-based imaging to map the spatiotemporal propagation of nonlinear strain in complex soft-matter environments [109]. Nonetheless, the resulting parameters should be interpreted as local rheological measurements rather than complete definitions of condensate-wide state [4,70,71].

Complementing probe-based microrheology, interfacial fluctuation analysis provides a non-perturbative readout of condensate interfacial mechanics by analyzing spontaneous boundary fluctuations. Flicker spectroscopy uses these fluctuations to infer interfacial tension and bending rigidity without embedded probes or externally applied forces [19,73]. Because condensate interfaces influence fusion, wetting, and phase coexistence, these measurements can relate interfacial mechanics to condensate behavior beyond bulk rheology [5]. Recent work has shown that flicker spectroscopy can be scaled to population-level measurements of condensate interfacial tension and bending rigidity under physiological imaging conditions (Figure 7Ci–vi) [73]. However, reliable fluctuation analysis still requires sufficiently well-resolved boundaries, appropriate temporal sampling, and models that remain valid for the condensate geometry and fluctuation spectrum under study [73].

### Controlled perturbation through microfluidic platforms

**Figure 8:**
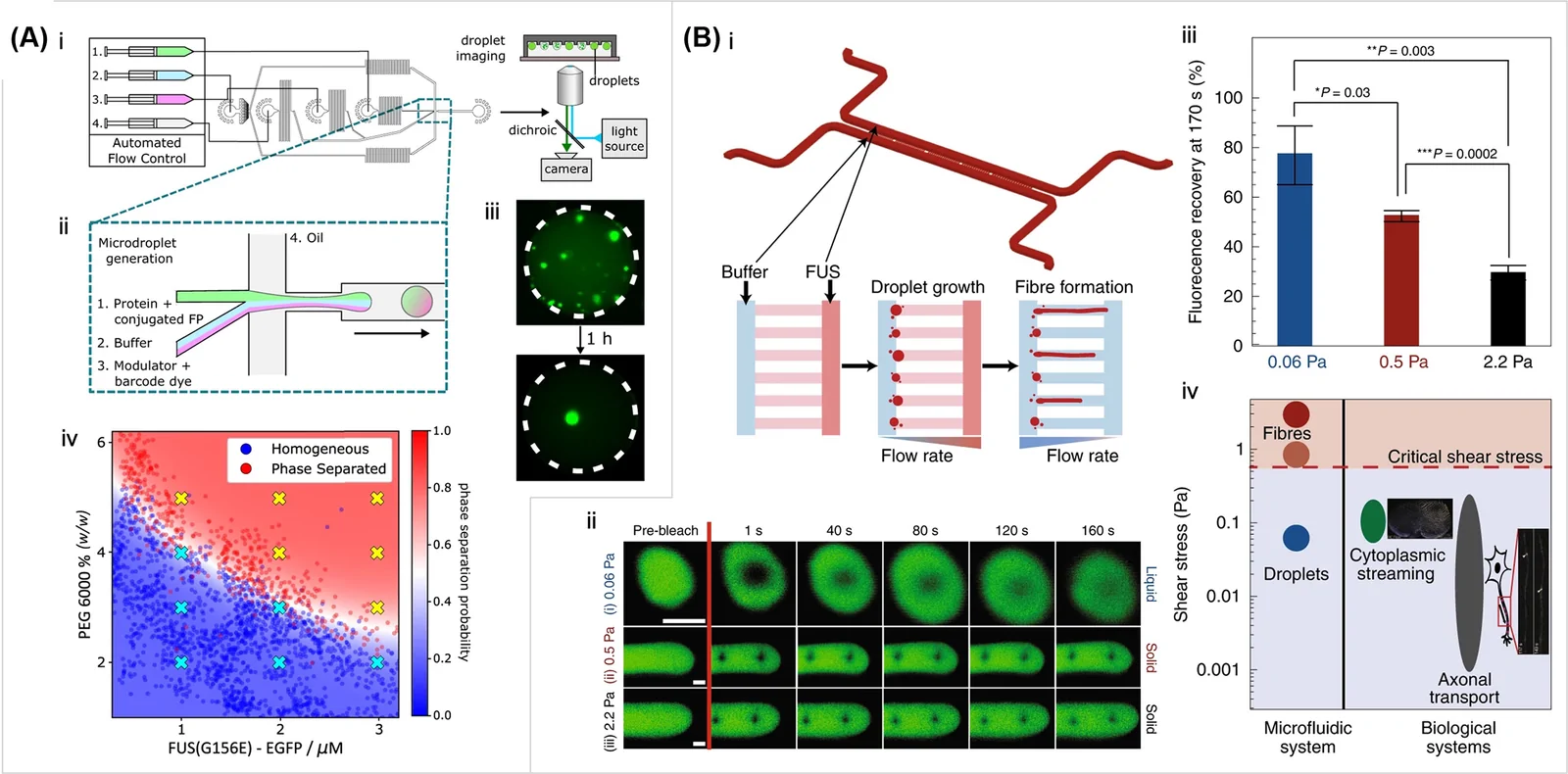
Microfluidic platforms for high-throughput phase mapping and controlled mechanical perturbation of condensates (**A**) PhaseScan microfluidics. (**i**) Automated flow-focusing microfluidic workflow for droplet generation and fluorescence readout. (**ii**) Laminar mixing of aqueous inputs before droplet formation. (**iii**) Time-resolved fusion of liquid condensates in microdroplets. (**iv**) Microdroplet-derived phase diagram showing the probability of phase separation across enhanced green fluorescent protein (EGFP)-FUS^G156E^ and polyethylene glycol (PEG) 6000 concentrations. Adapted from ref [75] under a Creative Commons Attribution CC-BY International License. Copyright 2022 by the authors. (**B**) Shear-flow microfluidics. (**i**) Device design and schematic of droplet growth and fiber formation under increasing flow-induced shear. (**ii**) Representative FRAP image series of condensates formed under different shear stresses. (**iii**) Quantification of fluorescence recovery after 170 s, showing reduced recovery with increasing shear stress. (**iv**) Comparison of the shear-stress regime explored in the microfluidic device with shear levels encountered in biological systems. Adapted from ref [74] with permission from the authors. Copyright 2020 by the authors.

Microfluidic platforms extend condensate probing by allowing controlled perturbation of composition, concentration, temperature, chemical environment, confinement, and flow, enabling the construction of multidimensional phase diagrams for phase-separating systems with far less sample and time than traditional plate assays [110]. One implementation of this concept is the PhaseScan platform, which allows high-resolution mapping of LLPS and quantitative measurements of small-molecule effects on phase behavior [75]. Using PhaseScan, changes in protein concentration and chemical environment can be systematically quantified to determine how biochemical conditions shift the boundaries for condensate formation and dissolution [75]. Beyond static phase mapping, PhaseScan enables high-throughput on-chip perturbation experiments, including controlled addition of ligands or nucleic acids, variation of ionic strength or macromolecular crowding, and time-resolved measurements of condensate assembly and dissolution dynamics (Figure 8Ai–iv) [75].

Key challenges nevertheless remain in translating droplet microfluidic approaches into routine high-throughput tools. Although droplet microfluidics can generate highly controlled and monodisperse compartments, conventional planar microchannel designs are often limited by relatively low droplet productivity, which restricts the scale of droplet generation and screening [111]. Three-dimensional inverse-colloidal-crystal channel architectures offer one solution, using densely parallelized micropores as micronozzles to increase droplet generation to more than 10 kHz [111]. However, increasing droplet production alone does not fully resolve the analytical bottleneck. Large-scale condensate assays also require robust droplet stabilization and handling, prevention of coalescence, suitable surface modification, and compatible downstream detection workflows [112]. More broadly, standardization and translation remain challenging because microfluidic performance is highly sensitive to device geometry, fabrication methods, materials, surface chemistry, and the integration of biological assay design with fluidic and instrumental expertise [113].

Microfluidic flow geometries have also been designed to probe interfacial mechanics under defined droplet deformations. For example, extensional-flow devices can trap and stretch droplets in cross-junctions to extract interfacial tension from steady or relaxing droplet shapes, offering an alternative to methods like MPA or fusion-kinetics measurements [114]. Recent platforms that actively control droplet coalescence inside channels improve reproducibility of stability tests and enable systematic mapping of coalescence thresholds across conditions relevant to condensates [115].

Beyond simple reconstituted droplet systems, microfluidics can also deliver controlled environmental perturbations in living systems. For instance, a microfluidic temperature-jump device applied to *Drosophila* embryos enabled rapid thermal perturbations while simultaneously quantifying nucleolar assembly kinetics of six different proteins [116]. Microfluidic shear-flow experiments have also shown that increasing flow-induced shear can drive condensates from droplet-like states toward fiber formation, accompanied by progressively reduced fluorescence recovery (Figure 8Bi–iii) [74]. This extends controlled perturbation to mechanical cues, allowing condensate maturation to be examined under defined shear stresses. The applied stress range overlaps with physiologically relevant regimes, linking controlled shear perturbation to aging-associated pathways in disease-relevant assemblies (Figure 8Biv) [74].

Across these approaches, mechanical characterization spans a continuum from observing spontaneous fluctuations to actively imposing force, deformation, or environmental change. Passive measurements constrain the material state under minimally driven conditions, whereas active manipulation and microfluidic perturbation reveal how that state responds when displaced from its resting condition. This distinction is informative because condensates with similar properties at rest may respond differently to applied stress or environmental change, exposing differences in relaxation, reversibility, or susceptibility to further state transition. Mechanical and perturbation-based readouts therefore provide complementary constraints on condensate material behavior, but because aging and LSTs often involve coupled changes in dynamics, structure, mechanics, and composition, their interpretation benefits from multimodal integration within the proposed readout framework.

## Multimodal integration for resolving condensate state transitions

Multimodal integration is a central component of the readout framework proposed in the present review. Biomolecular condensates often exhibit coupled changes in internal dynamics, viscoelastic response, interfacial mechanics, and molecular organization; single-readout classifications can therefore obscure how different aspects of condensate state evolve [5,9,117].

Accordingly, multimodal integration is particularly informative when complementary techniques constrain different aspects of the same transition, allowing apparent agreement or divergence between readouts to be interpreted [4,18]. For example, Schlüßler et al. combined fluorescence, optical diffraction tomography, and Brillouin microscopy in HeLa cells and found that the nucleoplasm exhibited a lower density but a higher longitudinal modulus than the cytoplasm, demonstrating that these two properties do not necessarily vary in parallel [83]. This distinction can be examined more directly by combining Brillouin microscopy with QPI, as demonstrated for FUS and hnRNPA1 condensates. In this system, Brillouin-derived parameters were used to characterize interaction- and dissipation-related properties, while QPI provided estimates of protein concentration and volume fraction, allowing mechanical changes to be considered in relation to condensate composition [118].

Multimodal integration also allows readouts obtained across different spatial and temporal scales to be related within the same transition. In one example, multimodal analysis of FUS aging combined MPA, FTIR, and SDM to probe condensate mechanics, structural reorganization, and spatially resolved internal dynamics during aging [10]. This integrated workflow showed that the LST was not spatially uniform, but instead initiated at the condensate surface and propagated inward, thereby linking macroscopic rheological changes with microscopic structural reorganization [10].

Correlative methods can also consolidate multiple material parameters within a single workflow. For instance, fluorescence recovery after probe-induced dewetting (FRAP-ID) combines AFM-induced dewetting with fluorescence recovery measurements to extract both viscosity and surface tension from the same condensate [119]. OpTiDDM provides another example, integrating local optical forcing with DDM to relate imposed deformation to the surrounding dynamic response [109]. These approaches illustrate a different form of integration, in which multiple material parameters are obtained within the same experimental context or an imposed perturbation is directly related to the resulting response, reducing ambiguity introduced by comparing measurements acquired under separate conditions.

Beyond direct combinations of experimental readouts, computational approaches can support multimodal workflows at the stages of interpretation, analysis, and acquisition. Coarse-grained molecular dynamics simulations of the plant RNA-binding protein PARCL (phloem-associated RNA-chaperone-like) have been used to compute residue interaction maps and identify candidate residues driving phase separation, followed by experimental validation *in vitro* and *in vivo* [120]. In addition, deep-learning analysis has enabled label-free detection of mutant huntingtin aggregates from transmitted-light images [121], while self-driving microscopy workflows have used neural-network-based event detection to trigger optimized multimodal imaging, including Brillouin readouts, at aggregation onset [122]. These studies show how computational approaches can connect prediction, event detection, and experimental measurement within a broader workflow for resolving condensate state transitions.

Across these examples, multimodal integration can reveal whether changes in composition, dynamics, structure, and mechanics occur in concert or become decoupled during a transition, helping distinguish similar endpoint states that arise through different transition pathways. Representative examples across reconstituted and cellular systems are summarized in Table 2.

**Table 2:** Representative integrated approaches for resolving condensate properties, organization, and state transitions across reconstituted and cellular systems

Integrated approach	Key insight enabled by integration	System/biological context	Reference
Fluorescence, optical diffraction tomography, and Brillouin microscopy	Enabled correlative measurement of refractive index, density, and Brillouin-based mechanics in intracellular condensate-like compartments, showing that phase-separated compartments do not necessarily exhibit the same trends in density and longitudinal modulus	Nucleoli, SGs, and polyQ aggregates in living cells	[83] (2022)
OpTiDDM	Coupled imposed strain, resistive force, and surrounding dynamics in one framework, linking force response to local material dynamics	Model polymer networks (chromatin mechanics)	[109] (2022)
MPA, FTIR, and SDM	Linked condensate mechanics, structural reorganization, and spatially resolved internal dynamics; showed that the FUS LST initiates at the surface and propagates inward	FUS condensates	[10] (2023)
Raman spectroscopy, solid-state NMR, and MPA	Combined spatial, structural, and mechanical readouts to support spatially non-uniform aging of FUS droplets, with a β-sheet-rich crust-like shell forming at the droplet surface	FUS condensates	[123] (2024)
Brillouin microscopy with QPI	Combined Brillouin and QPI readouts to quantify interaction strength, dissipation-related properties, and protein volume fraction / dry mass density in condensates	FUS and hnRNPA1-LCD condensates	[118] (2024)
FRAP-ID	Extracted viscosity and surface tension from the same condensate in a single correlative workflow	ELF3 condensates	[119] (2024)
Array-detector fluorescence correlation spectroscopy	Characterized condensates under flow, including droplet-size distributions and microfluidic flow profiling in microchannels	Tau condensates	[124] (2024)
QPI with ATRI	Enabled label-free measurement of multicomponent condensate composition and showed that fluorescent tags can alter condensate composition	Reconstituted multicomponent protein-RNA condensates including FUS, TAF15 RNA-binding domain (RBD), and poly(A) RNA	[22] (2025)
SRS microscopy	Enabled *in situ* quantitative imaging of secondary-structure changes within condensates, connecting phase separation with structural transition pathways	FUS and ATXN2 LCD condensates	[34] (2025)
Self-driving microscopy with intelligent Brillouin imaging	Used neural-network-based event detection to trigger targeted Brillouin imaging at aggregation onset, enabling automated biomechanical characterization of early aggregation events	Mutant huntingtin (mHtt)-polyQ aggregation model	[122] (2025)
Active machine learning (ML) with automated pipetting and autonomous confocal microscopy	Integrated automated formulation, imaging, and ML-guided sampling to efficiently map multidimensional condensate phase diagrams	Reconstituted poly-L-lysine/poly-L-aspartic acid coacervates	[125] (2025)
Coarse-grained molecular dynamics with experimental validation	Identified sequence determinants governing phase separation in the Phloem-Associated RNA-Chaperone-Like (PARCL) protein and validated these predictions experimentally *in vitro* and *in vivo*	PARCL RNA-binding protein	[120] (2025)
Stabilized real-time Brillouin microscopy with FRAP	Linked Brillouin shifts to FRAP immobile fractions in living-cell condensates, revealing percolation-like behavior and fractal internal organization beyond what FRAP alone resolved	Living-cell G3BP1 SGs and disease-associated superoxide dismutase 1, TAR DNA-binding protein (TDP-43), and FUS assemblies	[84] (2026)
High-throughput flow cytometry with confocal benchmarking	Resolved condensate phase behavior, molecular partitioning, and real-time exchange at single-droplet throughput, revealing reduced molecular dynamics upon aging	Nucleophosmin 1 (NPM1)-based condensate systems	[126] (2026)
Frequency-domain fluorescence lifetime imaging, optical tweezers, VPT, FRAP, and BCARS	Integrated lifetime, rheological, hyperspectral, and fluorescence readouts to show that dynamical arrest and amyloid-like transition begin preferentially at condensate interfaces, and that small-molecule-induced enhancement of condensate viscoelasticity weakens fibrillization propensity	Tau condensates	[80] (2026)
Cryo-electron tomography (Cryo-ET), single-molecule Förster resonance energy transfer, FRAP, and molecular dynamics simulations	Linked ultrastructural, conformational, and dynamic readouts to show that polar organizing protein Z (PopZ) forms a hierarchical filamentous condensate linked to wetting behavior and cellular function	PopZ condensates	[127] (2026)

## Outlook and Conclusions

### Measuring native transition pathways

A key future direction is to move from endpoint comparisons toward time- and space-resolved measurements of condensate transitions. Many studies compare condensates before and after aging, perturbation, or mutation. However, the more important questions are when a transition begins, where it initiates, how it propagates, and whether it remains reversible. Future measurements should therefore capture how dynamical, structural, compositional, and mechanical readouts change together, rather than treating each readout as an isolated endpoint. Extending this logic to cellular systems will be important for determining whether transition pathways observed in reconstituted condensates are preserved, modified, or buffered in native biological environments.

Achieving this goal does not necessarily require a completely non-perturbative method. Instead, it requires experimental designs that minimize avoidable perturbation, separate transition-dependent changes from experimental confounders, and explicitly report unavoidable perturbative factors. Fluorescent labels, embedded probes, surfaces, illumination, temperature history, sample age, and applied force may all influence the apparent timing, rate, or spatial pattern of condensate evolution. In this sense, pathway-resolved characterization shifts the purpose of condensate measurements from assigning a material label to understanding how a material state emerges and how confidently this process can be interpreted under defined experimental conditions [4,18].

### Integrated and adaptive technologies

Capturing transition pathways will require technologies that connect complementary readouts in time and space. Current methods can resolve individual dimensions of condensate state, while the multimodal examples summarized in Table 2 illustrate how the four major dimensions of condensate-state characterization (dynamics, structure, mechanics, and composition) can be integrated within a more interpretable framework. Future platforms could extend this logic by combining controlled microfluidic perturbations with real-time imaging, spectroscopy, or mechanical readouts to map condensate assembly, compositional remodeling, material-state changes, and functional responses under defined environmental conditions [75]. In cellular settings, such integrated platforms will need to balance readout sensitivity with minimal perturbation, so that endogenous condensate dynamics and functional outputs can be monitored without substantially altering the underlying phase behavior [18,19].

Adaptive microscopy and AI may further improve this process by complementing predefined acquisition and analysis strategies. On the acquisition side, event-triggered imaging could identify early signs of assembly, interfacial arrest, or aggregation and then switch to a higher-resolution, higher-speed, or mechanically informative modality, as illustrated by recent self-driving microscopy workflows that use neural-network-based event detection to guide Brillouin imaging [122]. On the analysis side, machine-learning tools can improve the robustness and throughput of dynamic readouts; for example, convolutional neural network (CNN)-based denoising of DDM intermediate scattering functions improves quantification from short movies or rapidly evolving dynamics [128]. Related active-learning workflows that combine automated liquid handling, autonomous imaging, and ML-guided sampling further show how automation can accelerate multidimensional condensate phase mapping [125]. However, AI-assisted workflows also inherit limitations from the microscopy data on which they are trained, including dataset shift, limited transferability, and reduced interpretability outside the training domain [129]. In condensate studies, these risks may be amplified by system-dependent morphology, aging-dependent dynamics, imaging-condition-dependent contrast, and analysis choices such as segmentation, ROI selection, and time-window selection. AI-assisted segmentation, event detection, denoising, and parameter selection should therefore be benchmarked against calibrated, interpretable readouts and quality-control metrics rather than treated as stand-alone outputs.

### Connecting state to function

The ultimate goal of most condensate studies is to link measured condensate-state changes to biological, pathological, or engineered functions. A readout is informative only if it helps explain how a specific state change affects the relevant functional outcome. Future studies should therefore move beyond reporting state descriptors and ask how these readouts connect to the relevant biological, pathological, or engineered context.

This connection is especially important in disease-associated systems, where dynamic condensates may mature into less reversible, solid-like, oligomeric, or fibrillar assemblies. Aberrant phase behavior of RNA-binding proteins such as FUS and TAR DNA-binding protein (TDP-43) has been linked to disease-relevant material states, but pathological relevance cannot be inferred from solidification alone [130]. Future studies need to distinguish which measured changes precede pathological conversion, which are downstream consequences of aggregation, and which may represent protective or reversible adaptations. This logic also underlies emerging condensate-modifying therapeutic strategies, which aim to identify interventions that restore or redirect aberrant condensate behavior rather than simply eliminate condensates as structures [131].

Although condensate formation and state changes are often associated with functional outputs, causality should not be inferred from appearance alone. A cautionary example comes from transcriptional condensates, where multivalent transcription-factor interactions correlate with the strength of transcriptional activation, but visible liquid-like droplet formation has neutral or inhibitory effects on activation [132]. This indicates that the molecular interactions driving condensation may be functionally relevant, while the visible droplet state is not the causal functional unit. Future studies should therefore ask which measured state variables, rather than condensate formation itself, regulate the functional process under investigation.

In engineered systems, the functional question is different but conceptually related. Here, assembly and LST pathways can be used as design variables to tune mechanical stability, responsiveness, release behavior, or long-term persistence. Recent biomaterials-focused work frames protein LLPS and LSTs as routes to both liquid condensates and solid aggregates, with potential applications in functional protein-based materials [11]. The aim is therefore to control how molecular organization and material state produce the desired function.

In conclusion, the next phase of condensate research should move from observing condensate formation toward understanding how condensates evolve and why those changes matter. Minimally perturbative methods are most powerful when they preserve transition pathways; integrated and adaptive technologies are most useful when they connect complementary observables at the right moment; and condensate-state measurements become most meaningful when they are linked to function. A major future direction is to extend minimally perturbative condensate-state probing into cellular environments, where condensates operate within native biochemical networks, crowded macromolecular surroundings, and spatially organized cellular architecture. This is pivotal for connecting protein phase transitions to biological functions, because condensate size, dynamics, structure, mechanics, and composition can differ markedly between reconstituted systems and cells. Label-free and minimally perturbative methods are required to investigate phase transitions under native physiological conditions while reducing the influence of fluorescent tags and artificial components. However, non-invasive measurements in cells remain challenging because endogenous condensates are often small, heterogeneous, dynamic, and embedded within complex biomolecular backgrounds. Despite these challenges, label-free techniques have been advancing on both the macroscale (e.g. Brillouin microscopy with QPI) and the nanoscale levels (e.g. Cryo-ET). Ultimately, condensate-state readouts should not end at classification; they should explain how molecular organization evolves into biological regulation, pathological failure, or material utility.

## Open Access

Open access for this article was enabled by the participation of the University of Sydney in an all-inclusive Read & Publish agreement with Portland Press and the Biochemical Society under an agreement with CAUL.

## Abbreviations

AFM: atomic-force microscopy
AI: artificial intelligence
ATRI: analysis of tie-lines and refractive index
BCARS: broadband coherent anti-Stokes Raman scattering
BLS: Brillouin light scattering
Cryo-ET: cryo-electron tomography
DDM: differential dynamic microscopy
DFC: dense fibrillar component
DRB: 5,6-dichloro-1-β-D-ribofuranosylbenzimidazole
FRAP: fluorescence recovery after photobleaching
FRAP-ID: fluorescence recovery after probe-induced dewetting
FTIR: Fourier-transform infrared spectroscopy
FUS: fused in sarcoma
G3BP1: Ras GTPase-activating protein-binding protein 1
GFP: green fluorescent protein
iSCAT: interferometric scattering
iSCORS: interferometric scattering correlation spectroscopy
LCD: low-complexity domain
LLPS: liquid–liquid phase separation
LST: liquid-to-solid transition
ML: machine learning
MPA: micropipette aspiration
NMR: nuclear magnetic resonance
OpTiDDM: optical-tweezers-integrating-differential-dynamic-microscopy
PARCL: phloem-associated RNA-chaperone-like
PF-QNM: PeakForce Quantitative Nano-Mechanics
QPI: quantitative phase imaging
ROI: region of interest
SDM: spatial dynamic mapping
SERS: surface-enhanced Raman spectroscopy
SGs: stress granules
SRS: stimulated Raman scattering
TDP-43: TAR DNA-binding protein 43
VPT: video particle tracking
